# The Burden of Mental Disorders in the world – a GBD 2021 analysis in the WHO regions

**DOI:** 10.1192/j.eurpsy.2025.1492

**Published:** 2025-08-26

**Authors:** V. Pinheiro, J. V. Santos

**Affiliations:** 1 ULS Matosinhos, Matosinhos; 2 University of Porto; 3 RISE-Health/Cintesis; 4 ULS Santo António, Porto, Portugal

## Abstract

**Introduction:**

The Global Burden of Disease (GBD) study has generated a plethora of worldwide data on mortality and disability, including the disease burden due to mental disorders, often amenable to interventions, essential for health planning.

**Objectives:**

This work aims to report the burden of mental disorders in disability-adjusted life years (DALYs), from 2001 to 2021, globally and in the six World Health Organization (WHO) regions.

**Methods:**

Retrospective descriptive study, using secondary data from the GBD 2021 Results Tool. Globally and for each of the six WHO regions, age-standardised DALY rates are reported and respective 95% uncertainty intervals, between 2001-2021, for both sexes and for males and females. All data analysis was performed using R version 4.0.5.

**Results:**

In 2021, the both-sex age-standardised DALY rate due to mental disorders was 1909.15 (1440.16 – 2437.88) DALYs per 100,000 globally, with great heterogeneity across regions: the Americas with 2379.96 (1786.30 – 3026.74) DALYs per 100,000, the highest burden, and the Western Pacific with 1517.45 (1159.48 – 1910.43) DALYs per 100,000, the lowest. Between 2001-2021, the global both-sex age-standardised DALY rate remained relatively stable and even decreased slightly until 2019 but a sharp increase occurred in 2020 and 2021. This pattern generally held up across regions, with the Americas consistently the region with the highest burden, followed by Eastern Mediterranean, Europe, Africa, South-East Asia and Western Pacific. The European region showed the largest increase in 2001-2021 (from 1895.12 (1435.12 – 2420.97) to 2162.03 (1609.92 – 2777.89)). The same pattern occurred in females across regions, but an important difference in males was observed, with the Eastern Mediterranean region presenting the highest burden in 2021 (2012.54 (1523.41 – 2569.42), after overtaking the Americas in 2008.

**Conclusions:**

The burden of mental disorders remained relatively stable between 2001-2019 with a sharp increase in 2020-2021 globally, and great heterogeneity between regions and some important differences between sexes. Besides opportunities for mutual learning, essential for health planning, cultural sensitivities and social/economic contexts can be important factors associated to these patterns: the COVID-19 pandemic may have been an important trigger for this sharper increase in burden. These results highlight the different patterns of disease burden due to mental disorders in the world and the need for tailored strategies.

**Disclosure of Interest:**

V. Pinheiro Conflict with: This article was supported by National Funds through FCT - Fundação para a Ciência e a Tecnologia, I.P., within CINTESIS, R&D Unit (reference UIDB/4255/2020)., J. V. Santos: None Declared

